# Effects of postcuring times on the trueness of 3D -printed dental inlays made with permanent resins

**DOI:** 10.1007/s00784-025-06319-z

**Published:** 2025-04-12

**Authors:** Yasemin Özden, Latife Altınok Uygun

**Affiliations:** Department of Restorative Dentistry, Faculty of Dentistry, University of Afyonkarahisar Health Sciences, Afyonkarahisar, 03030 Turkey

**Keywords:** Dental resin, Indirect restoration, Inlay, Surface accuracy, 3D printing

## Abstract

**Objective:**

The aim of this study was to evaluate the trueness of 3D-printed dental inlays fabricated using different permanent dental resins and subjected to distinct postcuring times.

**Materials and methods:**

A total of 180 inlay specimens were fabricated and divided into nine groups of 20 specimens each. The inlays were first designed using 3D design software (Ansys SpaceClaim) and then transferred to a 3D printer. Using LCD technology, 60 inlays were fabricated from Senertek P-CrownV3 Ceramic (Senertek) resin, another 60 inlays from VarseoSmile Crown Plus (Bego) resin and the final 60 inlays from Saremco Print Crowntec (Crowntec) resin. Each of these three groups was divided into three equally sized subgroups (*n* = 20) cured with 2,000, 4,000 and 6,000 flashes, respectively, using the Otoflash G171 device (NK Optik, Germany). Then, the specimens were scanned and digitised using an intraoral digital scanner, and their trueness was evaluated by superimposing the digital measurements on the reference design and calculating their root mean squares (RMSs) and total overlap ratios (TORs). MANOVA was used to compare the measurements, and Tukey’s test was utilised for the post hoc analysis.

**Results:**

Significant differences in trueness were observed among the inlays fabricated with different resin types (*p* < 0.001). The Crowntec resin had the lowest RMS (0.08 ± 0.018 mm) and the highest TOR (94.59 ± 2.49%), indicating the best trueness, while Senertek had the highest RMS (0.114 ± 0.017 mm) and the lowest TOR (80.15 ± 5.95%), reflecting the lowest trueness. The postcuring time also significantly affected the trueness of the inlays. The 6,000-flashes group had the lowest RMS (0.095 ± 0.02 mm), and the 4000-flashes group had the highest TOR (89.81 ± 0.5%). The interaction between the resin type and the postcuring time was significant for the TOR (*p* = 0.01), suggesting that trueness improvements are material dependent.

**Conclusion:**

Both the resin type and the postcuring time significantly influenced the trueness of the 3D-printed dental inlay restorations. The Crowntec resin consistently exhibited superior trueness, and the Senertek resin demonstrated the lowest trueness. The optimal postcuring time varied by material, but 4,000 flashes generally provided favourable trueness outcomes. These findings highlight the importance of selecting an appropriate resin and optimising the postcuring parameters to enhance the trueness of dental inlays, potentially improving their clinical fit and longevity.

**Clinical relevance:**

Appropriate resin selection and adherence to optimised postcuring protocols are essential for achieving clinically true 3D-printed restorations, ultimately improving their adaptations in dental applications.

## Introduction

Digital dentistry has revolutionised traditional workflows, making many restorative procedures more efficient and productive. Unlike conventional techniques, Computer Aided Design-Computer Aided Manufacturing (CAD/CAM) systems simplify the process and enable chairside restorations to be completed in a single session [1]. Digital inlay fabrication involves subtractive manufacturing using computer-aided milling, and additive manufacturing using 3D printing. Milling technologies can create restorations that are comparable to conventional restorations in terms of mechanical performance, biological properties and trueness by cutting a preformed block or disc [2]. However, they have the following disadvantages: inability to shape complex details, such as undercuts and carving geometries, and to produce more than one unit at a time. Moreover, subtractive manufacturing methods result in significant material waste during the cutting process, and the discarded material cannot be reused [3]. In contrast, additive manufacturing can create complex geometries by bonding or polymerising small-volume components layer by layer, and it has the potential to be far more productive than substractive manufacturing. Thus, dental resin manufacturers and dental clinicians are exploring more cost-effective 3D printing production methods for dental inlays [4].

Clinicians are considering the use of additive manufacturing techniques to produce resin–ceramic restorations in permanent dental treatments a new trend [5]. These materials are based on composites and filled with ceramic particles in varying amounts, depending on the manufacturer. It has been reported that 3D-printed resins meet the high dimensional stability and aesthetic requirements of dental restorations [6]. The primary difference in all photopolymerisation 3D printing technologies, from stereolithography apparatus (SLA) to digital light processing (DLP) and the latest LCD printing technology, lies in the light source and the imageing system [7, 8]. LCD printers use a colour LCD panel to create masks that block the 405 nm light emitted from an light-emitting diode (LED) back panel. The main difference between DLP and LCD 3D printing is the light intensity. Light intensity is an important factor in photopolymerisation, as it determines the printing speed and the degree of postcuring. Therefore, photopolymer resins suitable for DLP 3D printing can also be used in LCD 3D printing, provided that either the initiator amount is increased or the exposure time is extended [9]. The minimally invasive concept is widely applied and allows for the use of CAD/CAM technology in various types of fixed partial prostheses, such as inlays, onlays and crowns. Inlays are among the most complex CAD/CAM restorations due to their intricate margins and hidden areas. Several factors affect the success of CAD/CAM fixed partial prostheses, including the abutment design, the trueness of the digital impression, the settings of the design software and the trueness of the printing [10].

According to the International Organization for Standardization (ISO), *trueness* refers to the closeness of the agreement between the arithmetic mean of multiple test results and the true or accepted reference value. In other words, trueness indicates how close a measured value is to a known value or standard [11]. To test the trueness of an object, images obtained from the object are compared with those obtained from a high-precision industrial scanner, designated as the control group, and the size of the deviation between these image groups determines the degree of the object’s trueness [12]. *Reverse engineering* provides the digital reference needed to evaluate the trueness of an object. It involves obtaining all the information needed for the reproduction or redetailing of an object and creating a digital copy of the object through point clouds obtained by 3D scanners using coordinates of known points or optical systems [13]. Geomagic Control X (3D Systems, Rock Hill, USA) is the most commonly used reverse engineering software for trueness analysis in research studies and is specified in the ISO- 12836 standards [13, 14].

In digital dentistry, the trueness of 3D-printed inlays (i.e., how closely a fabricated inlay matches its original design) is critical for clinical success. Trueness is a key parameter influencing biomechanical compatibility, long-term durability and patient comfort. Previous studies have investigated the overall trueness of 3D-printed inlays. One study has shown that their trueness can be influenced by various factors, such as the production conditions, material type, layer thickness, degree of polymerisation shrinkage, size and power of the polymerisation unit, and thermal expansion or shrinkage during the polymerisation process [15]. However, the specific impact of postpolymerisation processes on trueness remains insufficiently explored.

It has been found that the degree of polymerisation is enhanced by the postcuring process [16]. Thus, this process influences the inlay’s final mechanical properties and residual monomer content. However, alterations in chemical bonds may lead to dimensional deformation and warping in thin areas, which can result in poor marginal fit, microleakage, secondary caries and increased plaque accumulation [17]. Bayarsaikhan et al. [18] demonstrated that extending the postcuring duration significantly improves flexural properties, Vickers hardness, biocompatibility and dimensional accuracy, they further found that elevated temperatures accelerate the polymerisation process, thereby enhancing its overall efficiency. However, further research is needed to better understand the effects of the postcuring duration, the postcuring device used and their impact on the trueness of the fabricated object.

In particular, no studies have evaluated the effects of different postcuring durations using the Otoflash G171 postcuring device (NK Optik, Baierbrunn, Germany) on inlay trueness. Although postcuring has been shown to affect mechanical properties and dimensional accuracy, its specific impact on the trueness of 3D-printed inlays remains unclear. Moreover, no studies have systematically evaluated how different postcuring durations using the Otoflash G171 device influence the trueness of inlay restorations. Given the increasing clinical adoption of 3D printing technologies, defining optimal postcuring protocols is essential for improving restoration accuracy and long-term performance. This study aimed to answer the following research question: How do different postcuring durations (2,000, 4,000, and 6,000 flashes) using the Otoflash G171 device affect the trueness of 3D-printed inlay restorations fabricated from different permanent dental resins? The null hypothesis (H₀) was that neither the type of resin nor the postcuring duration would significantly affect the trueness of the 3D-printed inlays.

## Materials and methods

The specimen size was determined using G*Power 3.1.97, indicating that at least 20 specimens were needed to achieve 80% statistical power with an effect size of 0.30 and a significance level (α) of 0.05. According to a previous study [19], a minimum specimen size of eight per group was considered sufficient. Thus, 180 inlay specimens were fabricated from three different resins (60 per resin). Then, each resin group was further divided into three groups of 20 specimens each that were postcured for different durations. To ensure consistency, all the specimens were prepared under standardised conditions.

To first obtain the reference dataset for the trueness analysis of the inlay specimens, the reference model was produced by initially scanning a mandibular posterior first molar phantom tooth (ANA- 4) using a (TRIOS ® 3, 3 shape, Copenhagen, Denmark). Then, this model inlay’s cavity design was optimised to preserve the tooth structure and to enhance the durability of the restorative material, and the geometric features of this design were carefully crafted using the 3D modelling and direct editing software Ansys SpaceClaim 2022R1 to ensure smooth boundaries and optimal material adaptation. The preparation dimensions included a 10° occlusal convergence angle, a 2 mm cavity depth and an isthmus width of one-third of the intercuspal width [20]. The inlay design, which included the support structures, was saved as an STL file and prepared for 3D printing using the ChituboxPro V1.4.1 software (Phrozen, Taiwan). The software sliced the STL file into layers suitable for printing, created the support structures manually and optimised the printing parameters. It then simulated a 168 × 90 × 190 mm^3^ printer tray on which the inlay specimens were designed, with their occlusal planes facing the tray and with a printing direction of 0°. The support structures were placed on the base of the digital models, with a contact point size of 0.60 mm. The print layer thickness was set to 50 μm.

Before printing was performed for all the groups, the 3D printer (Phrozen Sonic Mini 8 K MSLA) was calibrated and set according to the manufacturer’s specifications. Then, the STL files were transferred to the printer, and printing was carried out for each group of 60 inlays by resin type. To ensure standardisation and prevent polymerisation inconsistencies, the process was performed using the same resin batch, which was stored under controlled temperature and humidity conditions. The permanent dental resins used in this study are listed in Table 1. After the specimens were printed, they were cleaned, and the support structures were removed. To eliminate unpolymerised resin residues from the inlays, the inlays in the Senertek and Bego resin groups were first washed in 99% isopropyl alcohol for 3 min using an ultrasonic bath device. Then, they were placed in fresh alcohol and ultrasonically washed for 2 min more. In the Crowntec resin group, the inlays were washed by spraying them with 99% isopropyl alcohol, according to the manufacturer’s instructions. Then, all the specimens were wiped with cotton pellets and dried with compressed air. After this, the specimens were postcured using the Otoflash G171 device under 1.5 bar nitrogen for three different durations: Group 1, 2,000 flashes; Group 2, 4,000 flashes; and Group 3, 6,000 flashes. These specific postcuring durations were selected based on the manufacturer’s recommendations for optimal polymerization (Saremco = 2 × 2000 flashes, varseo smile crown plus = 2 × 1,500 flashes) and previous studies investigating the impact of postcuring on the mechanical and dimensional properties of 3D-printed resins [21–23]. It was aimed to determine the number of pulses above and below the curing times recommended by the manufacturers and to investigate the significance of the effect of this situation on the results. The Otoflash G171 operates with a flashlight wavelength range of 300–700 nm, with peaks at approximately 480 and 530 nm, ensuring effective polymerization of dental resins.

**Table 1:** General properties of permanent dental resins

Material	Resin Manufacturer	Fillers	Flexural strength	Viscosity	Matrix components
Bego (VarseoSmile Crown Plus)	Bego, Germany	30–50 wt%. (silanized dental glass, particle size 0.7 μm)	116 MPa	2.500–6.000 MPa	4.4'-isopropylphenol, ethoxylated and esterification products of 2-methylprop- 2enoic acid, dental glass silica, methyl benzoylformate, diphenyl (2,4,6-trimethylbenzoyl) phosphine oxide
Crowntec (Saremco Print-Crowntec)	Saremco, Switzerland	30–50 wt%. (silanized dental glass, pyrogenic silica, particle size 0.7 μm)	135 MPa	2.500–6.000 MPa	4.4'-isopropylphenol esterification products, ethoxylated 2-methylprop- 2enoic acid, dental glass silica, pyrogenic silica, initiators
Senertek (Senertek P-Crown V3 Seramic)	Senertek Türkiye	65 wt% nano ceramic	260 MPa	3500 MPa	No information available

**Fig. 1:**
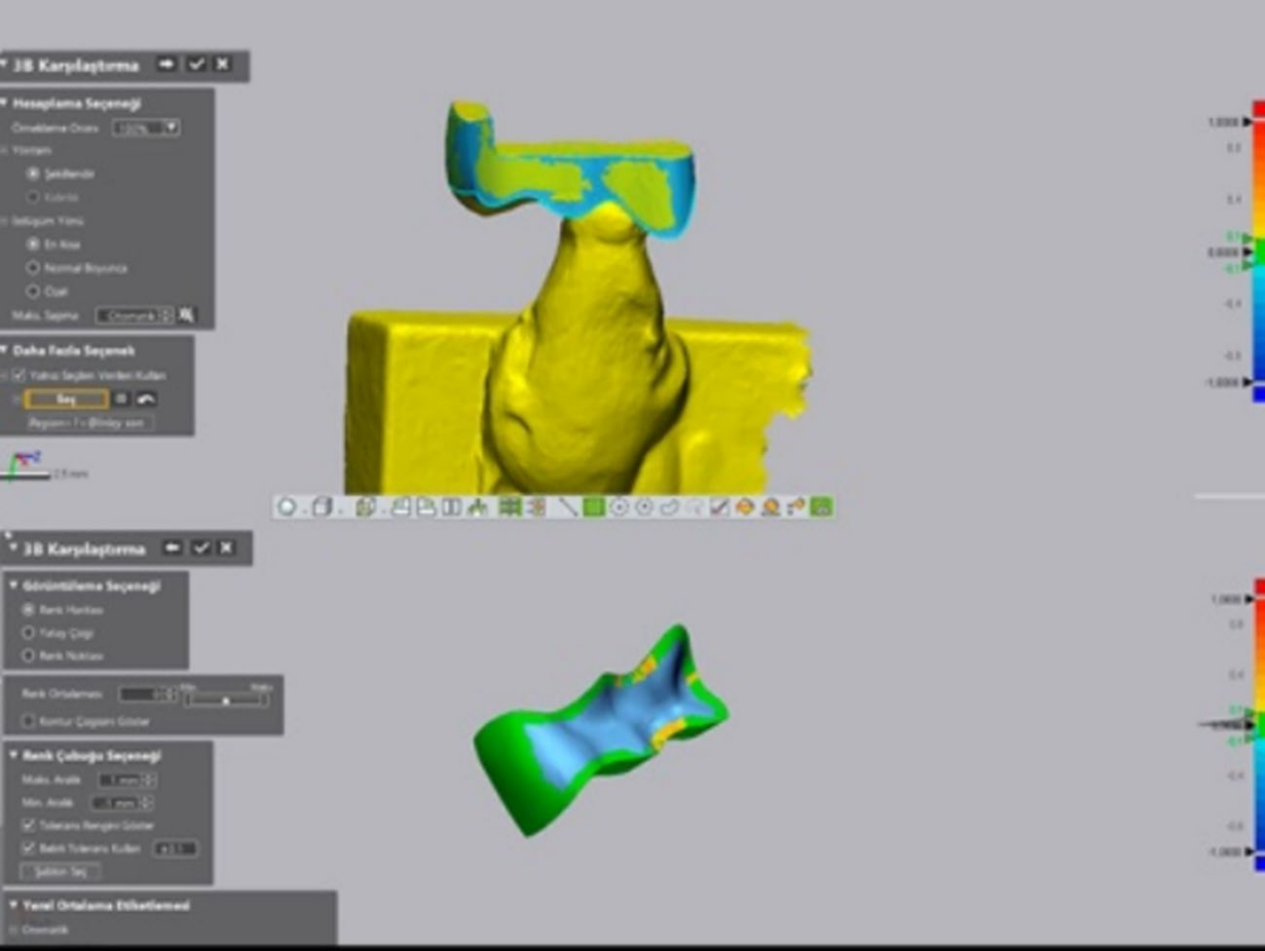
Digital overlapping operations using Geomagic Control X program

**Fig. 2:**
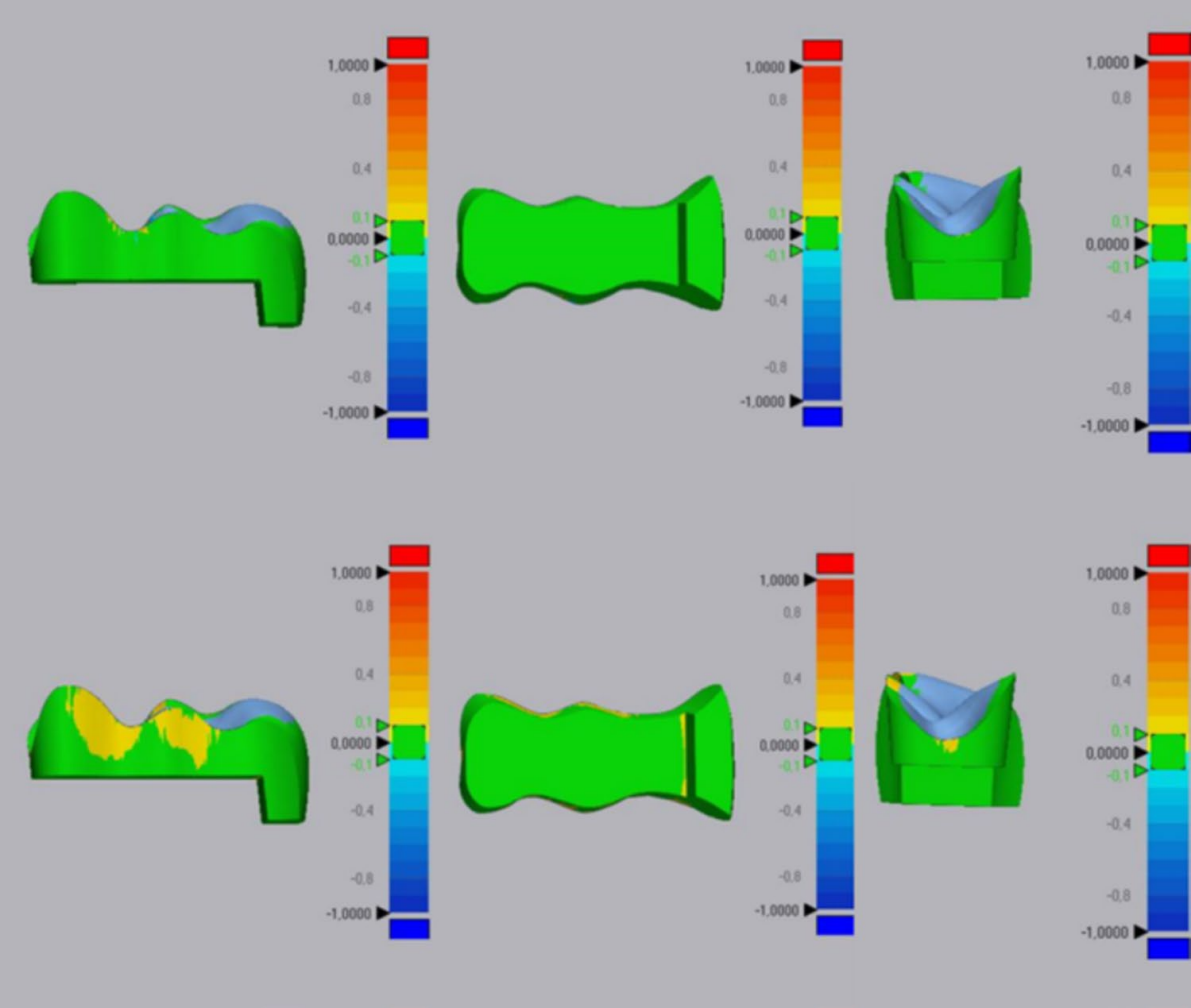
Inlay image in Geomagic Control X program

The 180 inlay specimens were then scanned using the Trios 3 scanner, and the digital files were exported to the STL format. Geomagic Control X was used to compare the reference design with the scanned specimens, employing the initial and best-fit alignments to assess 3D surface deviations using the root mean square (RMS) method. All the inlay surfaces, except for the occlusal surface, were included in the superimposition (Figs. 1 and 2). After the superimposition, the RMS and the total overlap ratio (TOR; %) results were obtained. Since variables such as 3D printing protocols and operator-dependent factors can introduce variability in experiment results, the same researcher (YÖ) rescanned 10% of the specimens and resuperimposed them on the reference design after two weeks, and recorded the results. The intraclass correlation coefficients were calculated to evaluate the consistency of the measurements. This process provided a quantitative assessment of the repeatability and reliability of the measurements, thereby serving as an important reference for this study’s outcomes [24]. All the statistical analyses were conducted using SPSS v24.0 (IBM), with statistical significance set at *p* < 0.05. Normal distribution was verified using the Shapiro–Wilk test. The effects of the resin type and the postcuring times on the trueness of the 3D-printed inlay restorations were analysed using Analysis of variance (MANOVA) and the Tukey HSD post hoc test.

## Results

**Fig. 3:**
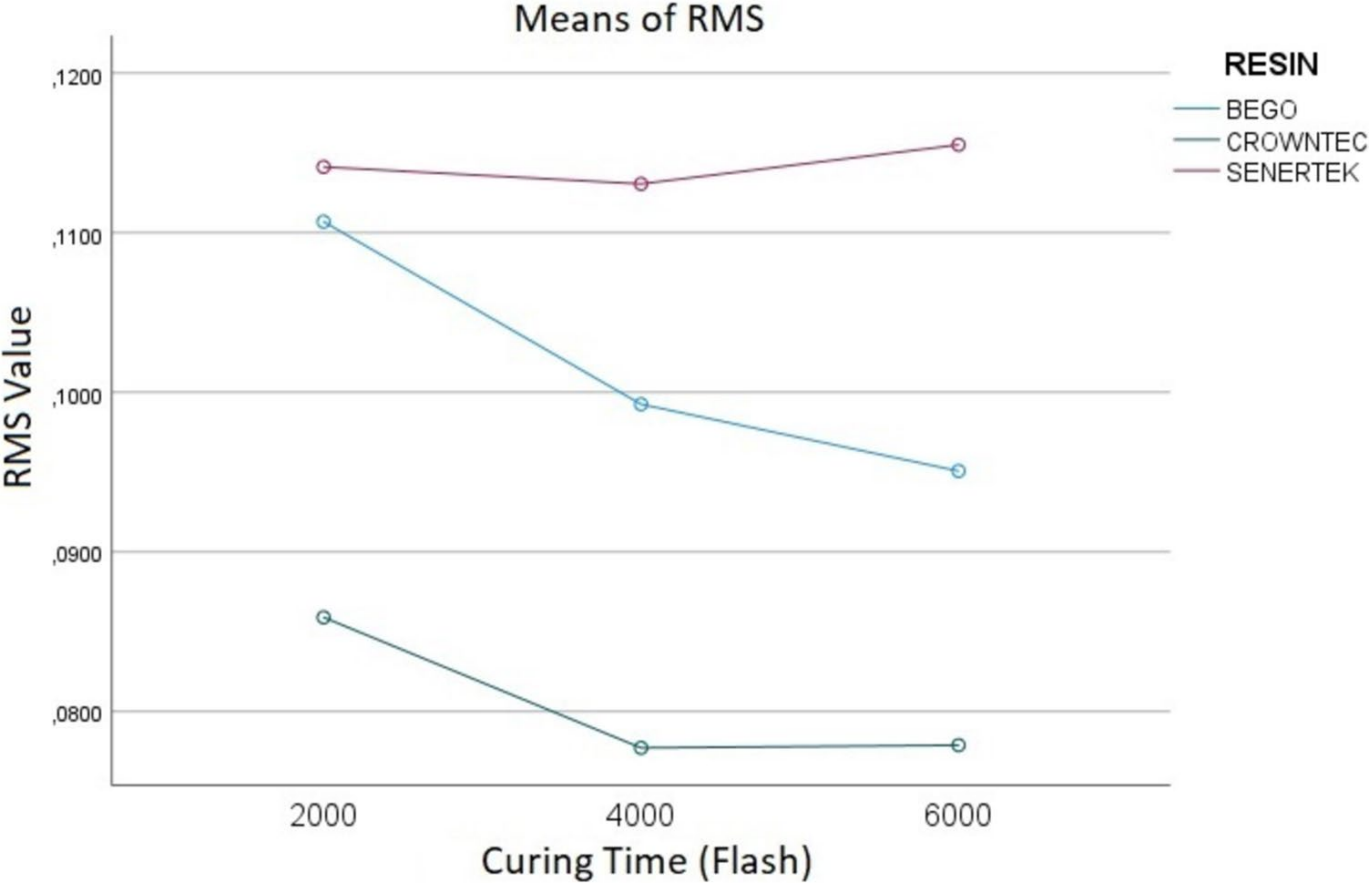
MANOVA Analysis of variance to compare the effect of resin and postcuring time on the RMS value of inlay restorations

**Fig. 4:**
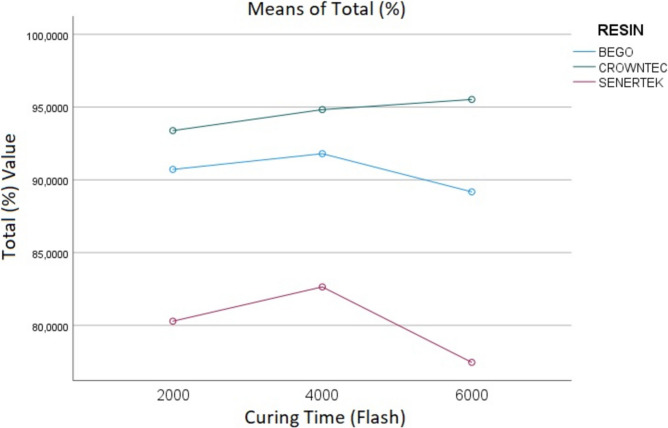
MANOVA Analysis of variance to compare the effect of resin and postcuring time on the TOR of inlay restorations

According to MANOVA, the types of resins used resulted in statistically significant differences in both the RMS (*p* < 0.001) and the TOR (*p* < 0.001). Similarly, the postcuring times led to statistically significant differences in both the RMS (*p* = 0.035) and the TOR (*p* = 0.004). However, the interactions between the resin types and the postcuring times did not result in statistically significant differences in the RMS values (p = 0.287; Fig. 3) but led to significant differences in the TORs (p = 0.01; Table 2; Fig. 4).

**Table 2:** Results of MANOVA Demonstrating the Effects of Resin Type, Post-Curing Time, and Their Interaction on RMS and Total Overlap Ratio

Source	Dependent Variable	Type III Sum of Squares	df	Mean Square	F	Sig
RESIN	RMS	0,03	2	0,01	58,03	< 0,001*
	Total Overlap Ratio (%)	6838,36	2	3419,18	205,33	< 0,001*
POST-CURING	RMS	0,00	2	0,00	3,43	0,035*
	Total Overlap Ratio (%)	186,84	2	93,42	5,61	0,004*
RESIN x POST-CURING	RMS	0,00	4	0,00	1,24	0,287
	Total Overlap Ratio (%)	223,245	4	55,81	3,35	0,010*
Error	RMS	0,05	181	0,00		
	Total Overlap Ratio (%)	3013,95	181	16,65		

* p < 0,05

Among the three resin groups, the highest RMS value was observed in the Senertek group (0.114 ± 0.017), followed by the Bego group (0.101 ± 0.017) and the Crowntec group (0.08 ± 0.018). Conversely, the highest TOR was observed in the Crowntec group (94.59 ± 2.49), followed by the Bego group (90.56 ± 3.75) and the Senertek group (80.15 ± 5.95).

Regarding the effect of the postcuring time on RMS, no statistically significant difference was found between Groups 2 and 1, and between Groups 2 and 3 (*p* = 0.099 and *p* = 0.946, respectively). However, a significant difference was observed between Groups 1 and 3 (*p* = 0.046). The mean RMS values and standard deviations (SDs) for the three postcuring time groups were as follows: Group 1, 0.103 ± 0.02; Group 2, 0.096 ± 0.02; and Group 3, 0.095 ± 0.02.

For the TOR, no statistically significant difference was found between Groups 1 and 2, and between Groups 1 and 3 (*p* = 0.137 and *p* = 0.440, respectively), whereas a significant difference was observed between Groups 2 and 3 (*p* = 0.005). The mean TORs and SDs were as follows: Group 1, 88.42 ± 0.51; Group 2, 89.81 ± 0.5; and Group 3, 87.54 ± 0.5.

The means and SDs of the RMSs for each resin type and postcuring time are presented in Table 3, while those of the TOs are shown in Table 4.

**Table 3:** The mean and standard deviation of RMS (mm) values for different resins and post-curing times in inlay restorations

Resin	Senertek	Bego	Crowntec	*p*
Post-Curing Times	Average (± Ss.)	Average (± Ss.)	Average (± Ss.)	
2000	0.120 ± 0,025^Aa^	0.111 ± 0.017^Aa^	0.086 ± 0.020^Ba^	< 0.001^*^
4000	0.116 ± 0.022^Aa^	0.101 ± 0.013^Aab^	0.078 ± 0.014^Ba^	< 0.001^*^
6000	0.118 ± 0.016^Aa^	0.095 ± 0.018^Bb^	0.077 ± 0.020^Ca^	< 0.001^*^
p	0.823^*^	0.005^*^	0.329^*^	

One-way ANOVA and post hoc Tukey test were performed for within-group comparisons. Different uppercase letters indicate significant differences within rows, while different lowercase letters indicate significant differences within columns (*p *< 0.05)

**Table 4:** The mean and standard deviation of Total Overlap Ratio (%) for different resins and post-curing times in inlay restorations

Resin	Senertek	Bego	Crowntec	*P*
Post-Curing Times	Average (%)	Average (%)	Average (%)	
2000	78.68 + 7.34^Aab^	90.26 + 3.44^Ba^	93.38 + 3.08^Ba^	93.38 + 3.08^Ba^
4000	82.31 + 4.00^Aa^	91.25 + 3.98^Ba^	94.41 + 2.70^Ca^	< 0.001^*^
6000	76.35 + 7.73^Ab^	88.44 + 5.86^Ba^	95.19 + 2.81^Ca^	< 0.001^*^
P	0.013^*^	0,114^*^	0.147^*^	

One-way ANOVA and post hoc Tukey test were performed for within-group comparisons. Different uppercase letters indicate significant differences within rows, while different lowercase letters indicate significant differences within columns (*p* < 0.05)

## Discussion

This study evaluated the effects of different resin types and postcuring times on the trueness of 3D-printed inlays. The findings showed that both of them significantly influenced the inlays’ trueness. The higher trueness was observed in the Crowntec 6,000 flashes group, and the lowest trueness values, in the Senertec groups. In general, 4,000 flashes appeared to provide favourable trueness outcomes across all three resin groups, but variations in the RMS and the TOR suggest that the optimal postcuring time may be material dependent. These results align with those of previous studies, such as Burjus et al. [25], who reported superior marginal fit for Crowntec compared to Senertec and Molinero et al. [26], who found that inlays fabricated using Crowntec exhibited superior trueness and adaptation compared to those fabricated using Bego. In the MANOVA results, the interaction between resin type and post-curing time was significant for TOR (p = 0.01) but not for RMS (p = 0.287). In Senertec groups, the RMS value increased as the post-curing time increased and showed the greatest loss of trueness. This may suggest that Senertec undergoes more polymerization shrinkage. Therefore, it can be said that this material is more sensitive to post-curing than other resins. While the post-curing time did not significantly affect the RMS value and trueness for Crowntec and Senertek, the longer post-curing time decreased the RMS value and increased the trueness for Bego. This may be due to the composition of Bego. In a study conducted by Karademir et al. [23], it was found that increasing the post-curing time of Bego improved its mechanical properties and this result was consistent with the result of the present study. Moreover, Bego demonstrated clinically acceptable production accuracy and simulated mid-term durability. Consequently, the null hypothesis that the resin type and the postcuring time would not significantly affect an inlay’s trueness was refuted.

In addition to the choice of material and the printer used in the additive manufacturing process, the choice of the impression-taking method can also affect the trueness of an indirect inlay [27]. The widespread adoption of digital impressions over traditional methods is due to several factors, including increased patient comfort during the impression-taking process, improved reproducibility, better data storage and easier fabrication. These advantages of digital impressions reinforce the notion that they are a viable alternative to traditional impressions [28]. In Silva et al.’s [29] comparison of digital and traditional measurement techniques in terms of dimensional accuracy, both techniques yielded clinically acceptable results, but the marginal fits of crowns produced with a digital intraoral scanner were superior to those of crowns produced with traditional silicone impressions. Therefore, intraoral scanners can be reliable alternatives to traditional indirect model scanners. The inlays produced in this study were scanned and digitised using an intraoral scanner, which eliminated the need for indirect scanners due to their small size and ability to perform standard scans without exposure to adverse intraoral factors. Then, the trueness of the inlays was evaluated.

Due to the higher resolution and lower cost of 3D printers using LCD technology compared to other 3D printing methods, they are becoming increasingly popular [9]. Tsolakis et al. [30] concluded that LCD printers can produce dental models reliably. A study that evaluated three LCD printers at different price points found that entry-level models achieved levels of trueness and precision that were comparable to those of their more expensive counterparts. One of the most recent studies on this topic concluded that Ultraviyole (UV) light postpolymerisation with LCD printing is an effective method of curing resins used for occlusal splints, dental models and temporary inlays [16, 31, 32]. Venezia et al. [31] compared the accuracy of four different 3D printers with distinct technologies—a DLP 3D printer, an entry-level LCD 3D printer and two SLA 3D printers—and the LCD printer demonstrated a performance level close to the clinical threshold in terms of trueness and precision for printed orthodontic models [31]. Tsolakis et al. [32] evaluated the trueness and precision of three different LCD printers and concluded that LCD 3D printers can be reliably used for model printing in dentistry and orthodontics. The results of previous studies are consistent with the findings of the present study.

Şahin et al. [33] assessed the mechanical properties of composite resins used in both additive and subtractive manufacturing and found that the flexural strengths of all the tested resins were acceptable for single-unit fixed inlays. Additionally, an in vitro study on the marginal gap and fracture resistance of 3D-printed permanent composite crowns revealed that implant-supported crowns made from the 3D-printed resin Crowntec exhibited superior marginal adaptation compared to those produced from millable restorative materials while maintaining similar fracture resistance. The 3D-printed implant-supported crowns also demonstrated marginal gap values below clinically acceptable thresholds and were capable of with standing occlusal forces in the posterior region [34].

The trueness of diagnostic and working inlay models produced with 3D printing technologies is influenced by factors such as the position of the model on the print bed, the layer thickness, the postproduction curing conditions, the type of printing technology and the model design [35–37]. Zhang et al. [38] compared the accuracies of 3D dental models obtained from different layers and concluded that the trueness of all the printers in their study was better than 50 μm. The trueness and surface quality of a 3D printer are closely related to the thickness of the layers added consecutively along the *z*-axis, with thicker layers resulting in increased errors. In this context, production was carried out with a layer thickness of 50 µm, following user instructions and referencing existing studies. In a study that evaluated the effect, on the bending strength, of resin specimens produced by positioning a 3D-printed production table at three different angles, the highest bending strength values were observed in the 0-degree orientation group [39]. Farag et al. [19] examined the marginal fit of crowns produced at different orientations (0°, 45° and 90°) using two permanent restoration resins (NextDent) with DLP and SLA printers and found that the 0° orientation provided the best marginal fit, followed by 45° and 90°.

To achieve optimal clinical alignment of the cavity surface with the inner surface of an inlay, it has been suggested in a study [40] that support structures be placed on the occlusal surface during the design process to minimise potential errors. However, after these support structures are removed, the rough surfaces may require polishing, which can impact the inlay’s trueness. Therefore, in the study, the support structures were positioned on the occlusal surface during the printing, and the rough surfaces created by these structures were excluded from the fitting process. Considering both the studies on the pressure direction and the clinical need for occlusal adjustments of the produced inlays after their cementation, it was decided that the angle between the inlays in this study and the production table should be 0° [19, 39]. It was concluded that the different polymerisation times and temperatures affected the dimensional trueness and the degree of polymerisation of the 3D-printed inlays [17, 41]. It has been suggested that selecting a final postcuring method appropriate to the type of 3D printer is crucial for ensuring the overall trueness of inlays, taking into account the possibility of shrinkage and the polymerisation dynamics [17].

Shin et al. [42] evaluated the effect of the final postcuring time on the dimensional stability of 3D-printed denture bases and reported that the dimensional stability improved as the postcuring time increased. Based on the study’s results, a minimum postcuring time of 30 min was recommended. The study also emphasised that dimensional changes after 3D printing mostly occur within the first day and that postcuring should be done immediately after printing.

Lee et al. [43], investigated the effect of the postcuring time using the LC- 3DPrint Box on the accuracy of the NextDent C&B material. They found that a postcuring time of 10 min—considerably less than the manufacturer’s recommended 30 min—resulted in significantly higher accuracy than the 20- and 30-min curing times [43]. As demonstrated, there is a significant relationship between the postcuring time and the production accuracy. Additionally, during inlay production with a photopolymer resin, the partially polymerised resin undergoes further hardening through the final postcuring process, which continues after the printing, thereby enhancing the stability of the printed product. An insufficient final postcuring time negatively affects the mechanical properties of the final product [44]. Postcuring with the Otoflash G171 in a nitrogen atmosphere provides a broad wavelength spectrum and supports various photoinitiator systems. Nitrogen polymerisation also prevents oxygen inhibition, thereby enhancing the polymerisation efficiency [45]. A study that analysed scanning electron microscope images of specimens cured with nitrogen gas found that these specimens exhibited smoother surfaces than those cured without nitrogen. Additionally, specimens produced with an LCD printer and subsequently cured with nitrogen demonstrated the highest Vickers hardness and conversion degree [17]. In the present study, the Otoflash G171 postcuring device, regarded as the gold standard for postcuring dental resins, was used in conjunction with a nitrogen gas-curing process. Currently, no studies in the literature have examined the effect of varying postcuring times with the Otoflash G171 on the trueness of permanent dental resins. Therefore, the results of this study are not directly comparable with those of other studies [46–48]. Deviations are generally expected during repeated scanning of the same specimens, primarily due to the sensitivity of the intraoral scanner.

The Trios 3 scanner has been reported to have an average precision of 14.1–14.9 μm in repeated scans under identical conditions, indicating minimal scanning errors [49]. Renne et al. [50] evaluated the accuracy of seven digital scanners: the CEREC Omnicam, CEREC Bluecam, Planmeca Planscan, Cadent iTero, Carestream 3500, Trios 3 and 3Shape D800 model scanners. They concluded that the scanners differed in terms of speed, trueness and precision, but the Trios 3 scanner provided the best combination of these three functionalities. For this reason, the Trios 3 scanner was selected in this study to digitise the specimens produced using a 3D printer.

When the average deviation between the measurement data and the reference model is minimal, the best-fit algorithm is used for registration, disregarding points not represented on the reference model and areas with significant deviations. This method employs reverse engineering software to automatically identify corresponding points between the printed model and the reference, forming a fixed surface for registration. High trueness and reliability in these processes are essential for the clinical applicability of the method [51–55]. Trueness was evaluated by digitally registering the reference data onto the data obtained from the LCD printer. To assess the 3D deviation values of the produced inlays, the RMS values provided by the software after optimal alignment, as well as the deviation values of the comparison points determined through the 3D comparison, were used.

Rossini et al. [47] suggested in their systematic review studies, which evaluated the measurement sensitivity and diagnostic trueness of digital models, that a measurement error of less than 200 µm is within clinically acceptable limits. However, the margin of error should be smaller for permanent inlays. In light of this suggestion, the best-fit algorithm was used to evaluate the trueness of the inlay specimens produced in the present study, with the maximum nominal value for colour spectra determined as ± 0.01 mm.

In this study, RMS values were analysed based on overlapping data from three different permanent dental resins and three different postcuring times. The results showed that the postcuring time did not lead to a statistically significant difference in RMS values for the Senertec and Crowntec resins. However, in the Bego group, a significant reduction in RMS was observed between 2,000 and 6,000 flashes, suggesting that extended postcuring improved the trueness of the inlays made with this resin. These findings indicate that the effect of the postcuring duration on trueness is material dependent, and the optimal postcuring time may vary based on the resin’s composition.

It is also thought that the increasing temperature in the postcuring device during long postcuring might have affected the ceramic structures of the resins in this study. An important limitation of this study is the lack of real-time temperature monitoring during the post-cure process. Temperature fluctuations may affect the polymerization dynamics and dimensional stability of inlays; especially resins containing ceramic fillers may exhibit different thermal expansion properties [56]. Future studies should include precise temperature control and monitoring systems to better understand the effect of temperature changes on trueness. Additionally, evaluation of the relationship between post-cure temperature and mechanical properties such as flexural strength and hardness may provide further information about the clinical relevance of post-cure conditions. Another limitation of this study is the limited number of materials evaluated. A more comprehensive comparison would require further investigation of different materials from various manufacturers. A third limitation of this study is its use of only one type of printer for each resin material, which limited the assessment of the effects of different printers (LCD, SLA and DLP) on the resins. The clinical significance of the trueness differences obtained in this study should also be considered. In the literature, acceptable trueness limits for indirect restorations are reported to be between 50–100 µm [43] When the RMS and Total Overlap Ratio values measured in this study were analysed, it was observed that although there were statistically significant differences between certain groups, all groups remained within clinically acceptable limits. In particular, inlays produced with Crowntec have higher truenesss3 - 4, indicating that this material may provide an advantage in terms of marginal and internal fit. However, long-term in vivo studies are required to evaluate the effect of differences on clinical performance. The final limitation of this study is its non-investigation of the impact of surface energy on bacterial adhesion, which warrants further research.

## Conclusion

Within the limitations of this study, the following conclusions are drawn:

– The trueness of the 3D-printed inlays was influenced by both the resin type and the postcuring duration.

– Among the resins tested, Crowntec exhibited the higher trueness, while Senertek showed the lowest trueness.

– Among all the resin groups, the highest trueness was generally observed at 4,000 flashes, but the differences in trueness between the postcuring times were material dependent.

One-way ANOVA and post hoc Tukey test were performed for within-group comparisons. Different uppercase letters indicate significant differences within rows, while different lowercase letters indicate significant differences within columns (p < 0.05).

## Data Availability

No datasets were generated or analysed during the current study.
